# Feeding back surveillance data to prevent hospital-acquired infections.

**DOI:** 10.3201/eid0702.010230

**Published:** 2001

**Authors:** R Gaynes, C Richards, J Edwards, T G Emori, T Horan, J Alonso-Echanove, S Fridkin, R Lawton, G Peavy, J Tolson

**Affiliations:** Centers for Disease Control and Prevention, Atlanta, Georgia 30333, USA. rpg1@cdc.gov

## Abstract

We describe the Centers for Disease Control and Prevention's National Nosocomial Infections Surveillance system. Elements of the system critical for successful reduction of nosocomial infection rates include voluntary participation and confidentiality; standard definitions and protocols; identification of populations at high risk; site-specific, risk- adjusted infection rates comparable across institutions; adequate numbers of trained infection control professionals; dissemination of data to health-care providers; and a link between monitored rates and prevention efforts.


### Feeding Back Surveillance Data To Prevent Hospital-Acquired Infections

**Robert Gaynes, Chesley Richards, Jonathan Edwards, T. Grace Emori, Teresa Horan, Juan Alonso-Echanove, Scott Fridkin, Rachel Lawton, Gloria Peavy, James Tolson, and the National Nosocomial Infections Surveillance (NNIS) System Hospitals**
Centers for Disease Control and Prevention, Atlanta, Georgia, USA

We describe the Centers for Disease Control and Prevention's National Nosocomial Infections Surveillance system. Elements of the system critical for successful reduction of nosocomial infection rates include voluntary participation and confidentiality; standard definitions and protocols; identification of populations at high risk; site-specific, risk-adjusted infection rates comparable across institutions; adequate numbers of trained infection control professionals; dissemination of data to health-care providers; and a link between monitored rates and prevention efforts.

According to a 1996 Institute of Medicine (IOM) report, preventable "adverse health events," a category defined as injuries such as medical errors (a failure of planned actions) and hospital-acquired infections caused by medical interventions, are responsible for 44,000 to 98,000 deaths per year at a cost of $17-$29 billion (1). The IOM report recommended immediate and strong mandatory reporting of medical errors and voluntary reporting of other adverse health events, suggesting that monitoring leads to reduction. A hallmark of monitoring any adverse health event is reporting the information back to those who need to know. We examine the value of feeding back information on hospital-acquired infections to reduce and prevent them.

### Hospital-Acquired Infections Surveillance Systems as a Model to Monitor and Prevent Other Adverse Health Events

Hospital-acquired infections affect approximately 2 million persons each year (2). Such infections have been monitored in the United States since the 1970s, and the monitoring is often a model for monitoring other adverse health events (3). Principles used in the surveillance of hospital-acquired infections are strikingly similar to those used in the continuous quality improvement process in manufacturing (4). Both systems emphasize changes at the system rather than individual level. Deming described two types of errors in manufacturing: special causes and usual causes. Special causes of error comprise only 5% to 10% of all errors; usual causes constitute the remainder. Similarly, only 5% to 10% of hospital-acquired infections occur in recognized outbreaks (4,5).

### Surveillance Systems for Hospital-Acquired Infections

Surveillance is defined as "the ongoing, systematic collection, analysis, and interpretation of health data essential to the planning, implementation, and evaluation of public health practice, closely integrated with the timely dissemination of these data to those who need to know" (6). The scientific value of surveillance as part of a hospital infection-control program was demonstrated most strongly in the landmark Study of the Efficacy of Nosocomial Infection Control (SENIC) (2). In that study, highly trained data collectors evaluated more than 338,000 patient records from a probability sample of U.S. hospitals to calculate infection rates. The hospitals' control programs were also evaluated. SENIC found that hospitals with the lowest nosocomial infection rates had strong surveillance and prevention programs. Other studies have suggested that surveillance also has a strong scientific basis. For example, the collection, calculation, and dissemination of surgeon-specific, surgical site infection (SSI) rates to surgeons were found to reduce SSI rates in all published studies (3,6-9).

During the last two decades, hospitals have established internal systematic monitoring of hospital-acquired infection rates. Monitoring with benchmarks external to those of a single hospital's surveillance system has also been suggested (10). A single hospital may use its own definitions, methods, and monitoring protocols. Developing a monitoring system with external benchmarks requires considerable additional effort.

To be successful, a multicenter monitoring system must satisfy three requirements: it must have a very clear purpose; it must use standard definitions, data fields, and protocols (including of cohorts or groups to be monitored and periods of data collection); and it must identify an aggregating inst itution to standardize definitions and protocols, receive the data, assess them for quality, standardize the approach to risk-adjusting the benchmarks, and interpret and disseminate the data.

### The NNIS System

The Centers for Disease Control and Prevention (CDC's) National Nosocomial Infections Surveillance (NNIS) system has been serving as an aggregating institution for 30 years. The NNIS system is a voluntary, hospital-based reporting

Address for correspondence: Robert Gaynes, Hospital Infections Program, Centers for Disease Control and Prevention, 1600 Clifton Road, Mailstop E55, Atlanta, GA 30333; fax: 404-639-6458; e-mail: rpg1@cdc.gov system established to monitor hospital-acquired infections and guide the prevention efforts of infection control practitioners. In 1999, 285 hospitals in 42 states participated in the NNIS system (11). All NNIS hospitals have ≥100 beds and, on average, are larger than other U.S. hospitals (median bed size: 360 versus 210); however, NNIS hospitals have a geographic distribution similar to all other U.S. hospitals. The NNIS system establishes a national risk-adjusted benchmark for nosocomial infection rates and invasive device-use ratios (12,13) by using uniform case definitions and data-collection methods and computerized data entry and analysis. To promote the latter, CDC provides infection control practitioners with 28 hours of training and sponsors a biennial conference.

Patients in intensive-care units (ICUs) are at high risk for nosocomial infections and since 1987 have been monitored in the NNIS system by site-specific, risk-adjusted infection rates according to ICU type (12). The risk-adjusted benchmark infection rates and device-use ratios are published annually for use by both NNIS and non-NNIS hospitals (12). (Internet address for NNIS SemiAnnual Report: http://www.cdc.gov/ncidod/hip/surveill/NNIS.htm).

### Data Quality

For an aggregating institution to assess the quality of data, meaningful surveillance definitions of adverse health events must be available. These definitions do not define clinical illness; rather, they are used for credible, consistent application across institutions. There is always a balance between the resources expended to find these cases and the value within the institution of using the collected data and comparing them to the external benchmarks. There is no single source of information that allows an infection control practitioner to accurately identify hospital-acquired infections. CDC definitions of nosocomial infections include clinical and laboratory information that requires training, counseling, and updating—tasks that are largely the responsibility of the aggregating institution. Several studies have examined attempts at shortcuts around the training and counseling components; all studies suggest that medical record abstractors perform very poorly compared with infection control practitioners in case-finding for nosocomial infections (9). Hospital-acquired infection case ascertainment is time-consuming, and the process is becoming more difficult with earlier discharge of patients and lack of agreement on methods of postdischarge surveillance (14,15). Progress in this area has been slow, and more efficient methods of case ascertainment are needed. Sands et al. have proposed using exposure to antimicrobial drugs as a sensitive method for finding cases of SSI in the postdischarge outpatient setting (16). Although this method is efficient, many institutions are unable to acquire antimicrobial-drug use data for outpatients who have recently undergone hospital surgical procedures. Finally, despite current difficulties, a recent study in NNIS hospitals suggests accurate case finding can be achieved (Table 1).

### Measuring Infection Rates: Endemic- or Epidemic-Disease Rates?

Surveillance measures the endemic-disease rate of nosocomial infection. Less than 10% of all nosocomial infections occur in recognized outbreaks (5). If an outbreak occurs in a hospital, it is often because one prevention strategy failed for a short period. The endemic-disease rate provides hospitals with knowledge of the ongoing infection risks of hospitalized patients when no recognized outbreaks are occurring. This rate represents 90% to 95% of all hospital-acquired infections (5). Thus, ongoing surveillance measures the endemic-disease rate. Unlike outbreaks, rates established by ongoing surveillance usually require that many problems be addressed to lower a high rate of infection.

Table 1. Estimates of accuracy of prospectively reported infections in nine NNIS Hospitals[a]

| Infection site | Predictive value positive (%) | Sensitivity (%) | Specificity (%) |
|---|---|---|---|
| Bloodstream | 87 | 85 | 98.3 |
| Pneumonia | 89 | 68 | 97.8 |
| Surgical site | 72 | 67 | 97.7 |
| Urinary tract | 92 | 59 | 98.7 |
| Other | 80 | 30 | 98.6 |

[a]Adapted from Emori TG, et al. Infect Control Hosp Epidemiol (17).

### Measuring Success in a Surveillance System

From 1990 through 1999, we examined risk-adjusted, hospital-acquired infection rates used by participating NNIS hospitals (18). We found that decreases in risk-adjusted infection rates occurred at all three body sites (respiratory tract, urinary tract, and bloodstream) monitored in ICUs (18). Substantial decreases in bloodstream infection rates occurred in medical (44%), surgical (31%), and pediatric (32%) ICUs (Figure). Decreases also occurred in other ICU types (Table 2)

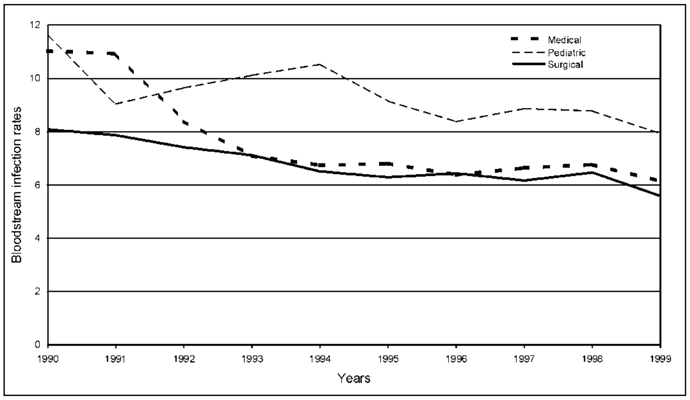

Figure. Trends in bloodstream infection rates by type of intensive care unit, National Nosocomial Infections Surveillance system, 1990-1999. Bloodstream infection rate is number of central line-associated primary bloodstream infections per 1,000 central line-days.

Table 2. Decrease in hospital-acquired infection rates, NNIS, 1990–1999

| Type of ICU | Bloodstream infection rate[a] (%) | Ventilator-associated pneumonia rate (%) | Urinary tract infection rate[b] (%) |
|---|---|---|---|
| Coronary | 43 | 42 | 40 |
| Medical | 44 | 56 | 46 |
| Surgical | 31 | 38 | 30 |
| Pediatric | 32 | 26 | 59 |

[a]Central line associated.
[b]Catheter associated.

and in infection rates at other sites (18). The reasons for these decreases are unknown, but several explanations are possible. First, the improvements seen in NNIS hospitals also reflect other national efforts to prevent infections (e.g., new research findings, prevention guidelines). Second, the U.S. health-care system has shifted away from hospital-based care. Some of the observed rate reductions could be attributable to this shift. However, a portion of these observed decreases likely represented true decreases in hospital-acquired infection rates in NNIS hospitals. Disseminating risk-adjusted, reliable infection rates within NNIS hospitals to infection control practitioners, patient care givers, and administrators was an essential part of NNIS efforts during the 1990s. By all reports, patient-care personnel began to perceive value in the data, relied on them for decisions, and altered their behavior in ways that may have reduced the incidence of nosocomial infections in NNIS hospitals. By changing the behavior of patient care givers, the NNIS approach to surveillance of nosocomial infections may have actually improved the quality of patient care. This report (18) demonstrated the value of the NNIS system as a model for preventing hospital-acquired infections (18).

### Critical Elements of a Surveillance System for Hospital-Acquired Infections

The NNIS elements critical for successful reductions in infection rates included 1) voluntary participation and confidentiality; 2) standard definitions and protocols; 3) defined populations at high risk (e.g., intensive care, surgical patients); 4) site-specific, risk-adjusted infection rates comparable across institutions; 5) adequate numbers of trained infection control practitioners; 6) dissemination of data to health-care providers; and 7) a link between monitored rates and prevention efforts, where patient-care personnel relied on the data to alter their behavior in ways that may have reduced the incidence of nosocomial infections (17).

### Challenges for the NNIS System's Future

Despite NNIS' success, many challenges remain. The IOM report recommends mandatory reporting of medical errors (1). Others have advocated public availability of such information. But achieving accurate data may be difficult if mandatory reporting and public availability of these data are required in all circumstances. These requirements heighten the need to assess the accuracy of self-reported data from institutions, a process that is difficult and expensive. The demand for publicly available data is particularly troubling. The NNIS Evaluation Study has suggested that, while data on nosocomial infections are generally accurately reported, sensitivity (underreporting of infections) was a more serious problem than other measures of accuracy such as predictive value positive or specificity (17). When the added pressure of publicly available data is added to a process that already has a tendency to miss cases of nosocomial infection, the possibility of serious underreporting of infections becomes cause for concern. Validating data is essential if data from performance measurement systems are to be credible.

All segments of the health-care community may not want or need the same data or the same level of detail in the data. Take the example of a consumer purchasing an automobile. The consumer rightly anticipates that the car will have a braking system that is safe and fully operational and thus would find the rate of errors for brake installation from the manufacturer of limited interest. This rate of error would be of vital interest to the manufacturer, however. Similarly, it is doubtful that regulators, payers, the public, or the health-care institution all want the same information with the same level of detail.

The medical marketplace is very dynamic. Surveillance must also be dynamic to keep pace with the changing environment. Improved methods of case ascertainment, especially with regard to postdischarge and outpatient surveillance, will be needed as more health care is provided outside the hospital. Improvement in measures of intrinsic and extrinsic patient risk factors will also be needed for improved risk adjustment. As computerization and integration of health care continue, these improvements will be possible. However, sound epidemiologic principles used by knowledgeable workers must guide use of the new technologies. A key to NNIS's success is infection control practitioners who use monitoring data to implement prevention activities. Any new system for preventing adverse health events will need to develop a cadre of professionals at the health-care facility to design and implement the prevention programs to promote patient safety and health-care quality (19).

Demonstrating the value of surveillance data to both the hospital's patient-care personnel and administration is essential. However, patient-care personnel must perceive value in the data; if they do, they will rely on the data for decisions and alter their behavior in ways that should reduce the incidence of nosocomial infections. By changing the behavior of care givers, surveillance of nosocomial infections or other adverse health events can improve the quality of patient care. However, SENIC suggested that only approximately one third of nosocomial infections are preventable (2). Better measures of adverse health events, including of nosocomial infections that are truly preventable, will make this monitoring more efficient and useful (20). Prevention measures will help move nosocomial infections from adverse health events to what the IOM described as medical errors (1). Solving the problem of medical errors still has its challenges.

Better understanding of the inner workings of the health-care delivery system to determine the root cause of errors is needed. Additionally, consistently good performers in a system where interhospital comparison of rates has been performed can identify the best practices. We are only beginning to understand the multiple prevention efforts of these high performers and how they differ from those of other institutions.

Despite the difficulties and challenges, application of epidemiologic principles can lead to success. A surveillance system to monitor hospital-acquired infections requires standardization, targeted monitoring, risk adjustment, trained professionals, and a link between the disseminated data and prevention efforts. A system such as the NNIS system with all these critical elements can be successful in preventing infections.

Dr. Gaynes is deputy chief, Healthcare Outcomes Branch, Division of Healthcare Quality Promotion, National Center for Infectious Diseases, CDC, and has been director of the NNIS system for 11 years. His main research interests are health-care acquired infections and antimicrobial-drug resistance.

### References

1. Institute of Medicine. To err is human. Washington: National Academy Press; 1999.
2. Haley RW, Culver DH, Morgan WM, Emori TG, Munn VP, Hooton TP. The efficacy of infection surveillance and control programs in preventing nosocomial infections in U.S. hospitals. Am J Epidemiol 1985;121:182-205.
3. Cruse PJE, Foord R. The epidemiology of wound infection: a 10-year prospective study of 62,939 wounds. Surg Clin North Am 1980;60:27-40.
4. Deming WE. Out of the crisis. Cambridge: Massachusetts Institute of Technology Center for Advanced Engineering Study; 1986.
5. Stamm WE, Weinstein RA, Dixon RE. Comparison of endemic and epidemic nosocomial infections. Am J Med 1981;70:393-7.
6. Ehrenkranz NJ. Surgical wound infection occurrence in clean operations. Am J Med 1981;70:909-14.
7. Condon RE, Schulte WJ, Malangoni MA, Anderson-Teschendorf MJ. Effectiveness of a surgical wound surveillance program. Arch Surg 1983;118:303-7.
8. Haley RW, Culver DH, Morgan WM, Emori TG, Munn VP, Hooton TM. Identifying patients at high risk of surgical wound infection: a simple multivariate index of patient susceptibility and wound contamination. Am J Epidemiol 1985;121:206-15.
9. Olson MM, Lee JT. Continuous, 10 year wound infection surveillance: results, advantages, and unanswered questions. Arch Surg 1990;125:794-803.
10. Sherertz RJ, Garabaldi RA, Kaiser AB, Marosal R, Berg RM, Gaynes RP, et al. Consensus paper on the surveillance of surgical wound infections. Infect Control Hosp Epidemiol 1992;13:599-605.
11. Emori TG, Culver DH, Horan TC, Jarvis WR, White JW, Olson DR, et al. National Nosocomial Infections Surveillance (NNIS) System: Description of surveillance methodology. Am J Infect Control 1991;19:19-35.
12. National Nosocomial Infections Surveillance System. Nosocomial infection rates for interhospital comparison: Limitations and possible solutions. Infect Control Hosp Epidemiol 1991;12:609-12.
13. Culver DH, Horan TC, Gaynes RP, and the National Nosocomial Infection Surveillance System. Surgical wound infection rates by wound class, operative procedure, and patient risk index in U.S. hospitals, 1986-90. Am J Med 1991;91(Suppl 3B):152S-157S.
14. Massanari RM, Wilkerson K, Streed SA, Hierholzer WJ Jr. Reliability of reporting nosocomial infections in the discharge abstract and implications for receipt of revenues under prospective reimbursement. Am J Public Health 1987;77:561-4.
15. Holtz T, Wenzel R. Postdischarge surveillance for nosocomial wound infection: A brief review and commentary. Am J Infect Control 1992;20:206-13.
16. Sands K, Vineyard G, Platt R. Surgical site infections occurring after hospital discharge. J Infect Dis 1996; 173:963-70.
17. Emori TG, Edwards JR, Culver DH, Sartor C, Stroud LA, Gaunt EE, et al. Accuracy of reporting nosocomial infections in intensive care unit patients to the National Nosocomial Infections Surveillance (NNIS) system: A pilot study. Infect Control Hosp Epidemiol 1998;19:308-16.
18. Centers for Disease Control and Prevention. Monitoring hospital-acquired infections to promote patient safety--United States, 1990-1999. MMWR Morb Mortal Wkly Rep 2000;49:149-52.
19. Scheckler WE, Brimhall D, Buck AS, Farr BM, Friedman C, Garibaldi RA, et al. Requirements for infrastructure and essential activities of infection control and epidemiology in hospitals: A consensus panel report. Am J Infect Control 1998;26:47-60.
20. Massanari RM, Wilkerson K, Swartzendruber S. Designing surveillance for noninfectious outcomes of medical care. Infect Control Hosp Epidemiol 1995;16:419-26.
